# Sp1-Induced FNBP1 Drives Rigorous 3D Cell Motility in EMT-Type Gastric Cancer Cells

**DOI:** 10.3390/ijms22136784

**Published:** 2021-06-24

**Authors:** Bo Kyung Yoon, Nahee Hwang, Kyu-Hye Chun, Yoseob Lee, Tatiana Patricia Mendes Duarte, Jae-Won Kim, Tae-Hyun Kim, Jae-Ho Cheong, Sungsoon Fang, Jae-woo Kim

**Affiliations:** 1 Department of Biochemistry and Molecular Biology, College of Medicine, Yonsei University, Seoul 03722, Korea; yoonbbk89@yuhs.ac (B.K.Y.); naheechel@gmail.com (N.H.); chunkh1@gmail.com (K.-H.C.); huru93@gmail.com (Y.L.); tai.duarte96@gmail.com (T.P.M.D.); jwk9394@yuhs.ac (J.-W.K.); taehyun@yuhs.ac (T.-H.K.); 2 Brain Korea 21 PLUS Project for Medical Science, Yonsei University, Seoul 03722, Korea; jhcheong@yuhs.ac; 3 Chronic Intractable Disease for Systems Medicine Research Center, College of Medicine, Yonsei University, Seoul 03722, Korea; 4 Department of Surgery, College of Medicine, Yonsei University, Seoul 03722, Korea; 5 Department of Biomedical Systems Informatics, College of Medicine, Yonsei University, Seoul 03722, Korea; 6 Severance Biomedical Science Institute, College of Medicine, Yonsei University, Seoul 03722, Korea

**Keywords:** gastric cancer, EMT, cell motility, FNBP1, Sp1

## Abstract

Cancer is heterogeneous among patients, requiring a thorough understanding of molecular subtypes and the establishment of therapeutic strategies based on its behavior. Gastric cancer (GC) is adenocarcinoma with marked heterogeneity leading to different prognoses. As an effort, we previously identified a stem-like subtype, which is prone to metastasis, with the worst prognosis. Here, we propose FNBP1 as a key to high-level cell motility, present only in aggressive GC cells. FNBP1 is also up-regulated in both the GS subtype from the TCGA project and the EMT subtype from the ACRG study, which include high portions of diffuse histologic type. Ablation of FNBP1 in the EMT-type GC cell line brought changes in the cell periphery in transcriptomic analysis. Indeed, loss of FNBP1 resulted in the loss of invasive ability, especially in a three-dimensional culture system. Live imaging indicated active movement of actin in FNBP1-overexpressed cells cultured in an extracellular matrix dome. To find the transcription factor which drives FNBP1 expression in an EMT-type GC cell line, the FNBP1 promoter region and DNA binding motifs were analyzed. Interestingly, the Sp1 motif was abundant in the promoter, and pharmacological inhibition and knockdown of Sp1 down-regulated FNBP1 promoter activity and the transcription level, respectively. Taken together, our results propose Sp1-driven FNBP1 as a key molecule explaining aggressiveness in EMT-type GC cells.

## 1. Introduction

Advances in genomic technology have brought new ideas in understanding cancers by uncovering genetic heterogeneity and resulting phenotypical differences among patients [1]. Gastric cancer (GC) is mostly adenocarcinoma; however, it is known for its great differences between subtypes [2,3]. As an effort, The Cancer Genome Atlas (TCGA) project defined four molecular subtypes of GC; Epstein–Barr virus (EBV), microsatellite instability (MSI), genomically stable (GS), and chromosomal instability (CIN) [4]. Meanwhile, the Asian Cancer Research Group (ACRG) analyzed clinically relevant molecular subtypes of GC; MSI, epithelial-to-mesenchymal transition (EMT), microsatellite-stable/TP53- (MSS/TP53-), and MSS/TP53+ GC [5]. The GS subtype from the TCGA project and the EMT subtype from the ACRG study are not identical but have similarities in having high portions of diffuse histologic type with worse prognosis [6]. We also have defined a stem-like subtype of GC patients with mesenchymal features which bring chemo-resistance and low survival rate [7]. Unfortunately, there is no targeted therapeutics available for this subtype with a high degree of EMT, requiring thorough investigation of the molecular factors contributing to the aggressiveness of this specific subtype compared to others.

Formin-binding protein 1 (FNBP1) is a member of the formin-binding protein family. Formin-binding proteins are modulators of formins [8] which are involved in actin polymerization, especially at the fast-growing end [9,10]. Among 32 mammalian formin-binding proteins [11], FNBP1 is reported as a curvature-sensing protein [12,13], membrane tension sensor protein [14], and membrane curvature regulator [15]. Although the studies on FNBP1 in cancer cells are marginal, FNBP1 has been reported to have a role in invadopodia formation in breast and bladder cancer [11,16,17]. The function and clinical relevance of FNBP1 in GC, however, have not yet been identified especially in the view of the critical determinant of the specific subtype, in the certain cell culture condition. 

Specificity protein 1 (Sp1), as one of the earliest transcription factors to be identified, regulates various cancer-related genes [18,19]. As a long-standing therapeutic target in cancer, ref. [20] Sp1 is known as a negative prognostic marker in GC [21,22]. Since there is a wide range of differences in FNBP1 expression level in GC cell lines, unlike most other cancer cell lines of different organs, the FNBP1 promoter region is re-visited to identify its transcription driver. Here, we first report that FNBP1 is a determinant of cell motility in EMT-type GC with Sp1 as its transcription inducer. In an effort to identify the critical factors contributing to the aggressive behavior of gastric cancer, we found that FNBP1 expression is correlated with the worst prognosis. FNBP1 drives cell motility, which is essential for the invasive or metastatic characteristics of the cancer. Sp1 was found to regulate FNBP1 expression for this aggressive behavior, indicating that the Sp1-FNBP1 pathway could be a new therapeutic target to inhibit metastasis in EMT-type GC.

## 2. Results

### 2.1. FNBP1 Is Highly Expressed in Aggressive Subtype of GC Patients

**Figure 1:**
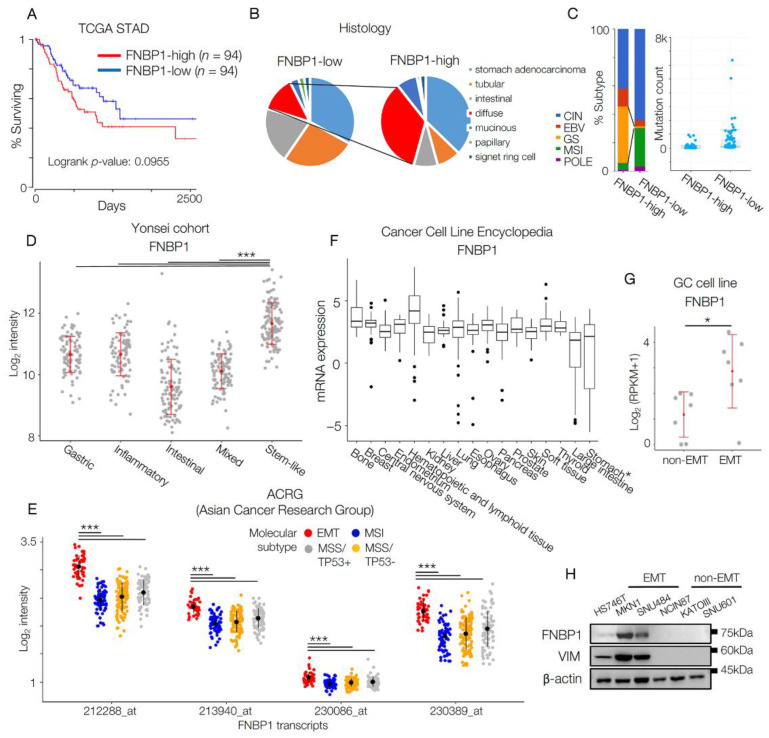
Aggressive subtype of GC cells selectively express FNBP1. (**A**) Kaplan–Meier plot for FNBP1-high group (*n* = 94) and FNBP1-low group (*n* = 94) in the TCGA cohort for stomach adenocarcinoma patients. Logrank *p* = 0.0955. (**B**) Pie chart displaying the distribution of histologic subtypes of FNBP1-high group (*n* = 94) and FNBP1-low group (*n* = 94) in the TCGA STAD. Diffuse-type is marked as red. (**C**) Bar graph displaying the distribution of genetic subtypes of FNBP1-high and low patients (left). GS-type is marked as yellow. Mutation count is also compared (right). (**D**) Transcriptome data of tumors of gastric cancer patients (*n* = 497) in Yonsei cohort was analyzed. The expression level of FNBP1 is compared according to the subtype: mixed (*n* = 99), gastric (*n* = 89), stem-like (*n* = 117), intestinal (*n* = 102), and inflammatory (*n* = 90). (**E**) Transcriptome data of tumors of gastric cancer patients (*n* = 300) in the ACRG cohort (GSE66229) was analyzed. The expression levels of FNBP1 transcripts are compared according to the subtype: EMT (*n* = 46), MSI (*n* = 68), MSS/TP53+ (*n* = 107), and MSS/TP53- (*n* = 79). (**F**) FNBP1 expression level from CCLE (Cancer Cell Line Encyclopedia) was marked according to its origin. (**G**) RNAseq data of GC cell lines were analyzed. FNBP1 level is marked according to the subtype. (**H**) Immunoblot of FNBP1 in GC cell lines. Data represent mean ± SD. * *p* < 0.05; *** *p* < 0.001; two-tailed *t*-test for (**G**); *p*-value was adjusted with Benjamini–Hochberg false discovery rate method for (**D**,**E**).

To evaluate the expression level of FNBP1 and its clinical relevance, the TCGA cohort for stomach adenocarcinoma (STAD) was first visited. Among 440 patients, we grouped FNBP1-high patients (*n* = 94) and FNBP1-low patients (*n* = 94) and analyzed the molecular and clinical differences. First, FNBP1-high patients showed worse survival, although not statistically significant (*p*-value = 0.0955) (Figure 1A). It is noteworthy that the FNBP1-high group shows a higher portion of diffuse histologic type (Figure 1B). According to Lauren’s classification [23], there are two main histologic types, intestinal and diffuse types. Compared to the intestinal-type, diffuse-type GC cells show a high degree of stromal infiltration with the loss of cell adhesion [24,25]. Diffuse-type, which is more prone to metastasize than the intestinal-type, is considered a worse prognostic marker [24,26]. According to the molecular subtypes characterized by the TCGA project [4], the GS subtype is resistant to chemotherapy with the worst prognosis [6]. Interestingly, the number of GS subtype tumors was much higher in the FNBP1-high group, supported by high mutation counts in the FNBP1-low group (Figure 1C). 

As an effort to understand GC heterogeneity and establish therapeutic regimens based on different molecular characteristics, we have previously grouped GC patients into five subtypes (mixed, gastric, stem-like, intestinal, and inflammatory) according to a validated molecular scheme, and the stem-like subtype was figured out to have the worst prognosis without the benefits of adjuvant chemotherapy [7]. Consistent with the data in the TCGA STAD, FNBP1 was significantly up-regulated in the stem-like subtype (Figure 1D). Meanwhile, in the ACRG cohort, FNBP1 was highly enriched in the EMT-subtype tumors (Figure 1E). 

To validate the findings in vitro, the expression level of FNBP1 in GC cell lines was evaluated. Unlike other organs which show relatively uniform up-regulation of FNBP1 across the cell lines, stomach and large intestine cancer cell lines revealed a wide range of FNBP1 expression levels, indicating its potential to be the subtype-specific marker (Figure 1F). Narrowing down to GC cell lines, the expression level of FNBP1 was higher in EMT cell lines than in non-EMT cell lines (Figure 1G,H). 

### 2.2. RNA-seq Analysis in shFNBP1 Stable Cell Line Shows Changes in Cell Periphery

**Figure 2:**
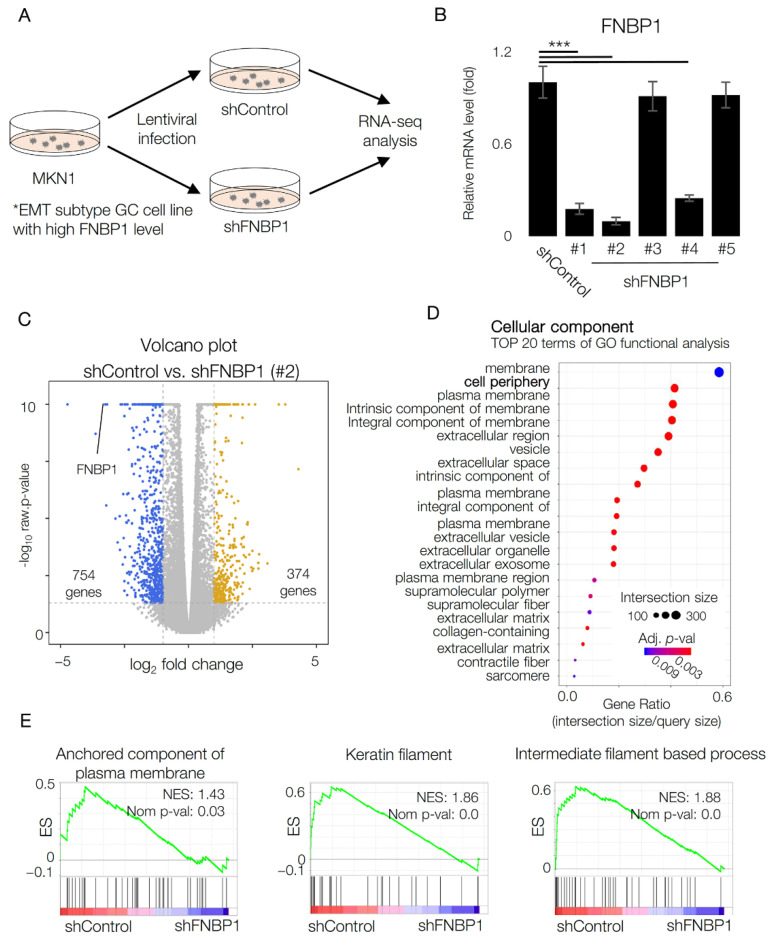
Transcriptome analysis reveals change in cell periphery. (**A**) Diagram to explain the experimental procedure. (**B**) qRT-PCR results to compare mRNA level of FNBP1 after lentiviral infection and puromycin selection. (**C**) Transcriptomic profile of MKN1 was analyzed via mRNA sequencing analysis after lentiviral infection. Genes with fold change greater than 2 and raw *p*-values smaller than 0.05 are marked in yellow. Genes with fold change less than 2 and raw *p*-values smaller than 0.05 are marked in blue. (**D**) Gene ontology enrichment analysis with differentially expressed genes. Top 20 terms of GO functional analysis in terms of cellular component are shown. The size of dots indicates intersection size, and the color denotes adjusted *p*-value. (**E**) GSEA for GO:0046658 anchored component of membrane (left), GO_CC_KERATIN_FILAMENT (middle), and GO_BP_INTERMEDIATE_FILAMENT_BASED_PROCESS (right). Data represent mean ± SD. *** *p* < 0.001; two-tailed *t*-test.

Next, we generated an FNBP1 knockdown stable cell line for further transcriptome analysis and phenotypic assays. After confirming selective expression of FNBP1 in EMT GC cell lines, we transduced cells with shRNA-encoding lentivirus with five different sequences expected to target FNBP1 expression (Figure 2A) in MKN1 which showed the highest expression of FNBP1 (Figure 1H). FNBP1 mRNA level was measured by qRT-PCR after lentiviral transfection. The MKN1 stable cell line with the lowest FNBP1 level was selected for further RNA-seq analysis (Figure 2B). 

We performed a volcano plot analysis to identify transcriptomic changes which resulted from FNBP1 knockdown. The volcano plot analysis indicated that the transcriptomic profile is distinctly separated by FNBP1 knockdown with 374 genes up-regulated and 754 genes down-regulated (Figure 2C). Up-regulation was defined by fold change greater than 2 with a raw *p*-value smaller than 0.05, and down-regulation was defined by fold change less than 2 with a raw *p*-value smaller than 0.05. We next conducted gene ontology enrichment analyses with differentially expressed genes (Figure 2D). In terms of cellular component, genes related to the plasma membrane and cell periphery were significantly altered upon FNBP1 knockdown. Consistent with the gene ontology analysis, the gene set related to the anchored component of the plasma membrane was significantly enriched in MKN1 control cells compared to FNBP1 knockdown cells in gene set enrichment analyses (GSEA) (Figure 2E). Given that FNBP1 is expressed in EMT GC cell lines with high invasive capacity, GSEA also revealed changes in cell motility-related pathways (Figure 2E) [27,28]. 

### 2.3. Ablation of FNBP1 Results in the Loss of Invasion Capacity

**Figure 3:**
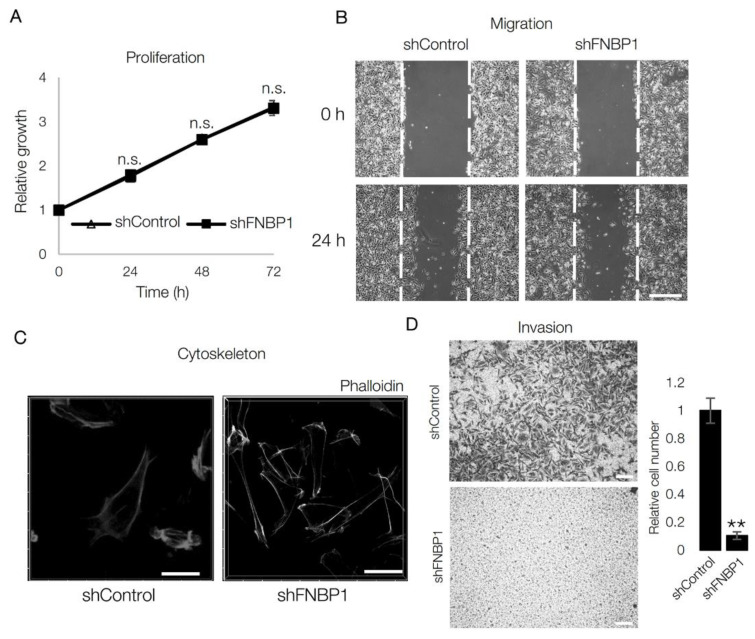
Ablation of FNBP1 results in the loss of invasion capacity. (**A**) Proliferation assay was done in MKN1 stable cell lines. “n.s.” indicates not significant (*p* > 0.05). (**B**) Migration capacity was compared by analyzing the area re-filled with cells after 24 h. Dotted lines denote the site of the scratch wound. Scale bar: 30 µm. (**C**) Confocal image shows fibrous actin distribution after fixed cells are stained with phalloidin. Images are 3D reconstructed. Scale bar: 40 µm. (**D**) Invasion assay was performed with stable cell lines. Cells were counted after 8 h of incubation. Scale bar: 50 µm. Data represent mean ± SD. ** *p* < 0.01; two-tailed *t*-test.

After analyzing changes in transcriptomic signatures resulting from the loss of FNBP1, we characterized cell behaviors in response to FNBP1 knockdown. Phenotypic assays are performed in MKN1 stable cell line expressing FNBP1-targeting shRNA (Figure 2A). First, consistent with the transcriptomic finding that there is no change in cell cycle-related gene set (Table 1), FNBP1 knockdown did not affect the proliferation rate of the cells (Figure 3A). Next, we investigated the migration rate and cytoskeletal change. Since FNBP1 knockdown induced mRNA profile change regarding plasma membrane, cell periphery, and filament-based process, we hypothesized a significant change in migration rate and cytoskeletal system. However, unexpectedly, there was no change in migration rate in the monolayer culture (Figure 3B). Stress fibers are actin bundles that enable contractile movement to the extracellular matrix and are another character of EMT-type GC such as MKN1 [29,30]. As in the migration assay, loss of FNBP1 did not alter the stress fiber formation in MKN1 (Figure 3C). However, FNBP1 knockdown significantly decreased the invasive capacity of MKN1, indicating its lack of ability to penetrate the extracellular matrix (Figure 3D). Thus, ablation of FNBP1 selectively inhibited cellular invasion through a dense extracellular matrix [31,32]. 

### 2.4. FNBP1 Increases Cell Motility in Extracellular Matrix

**Figure 4:**
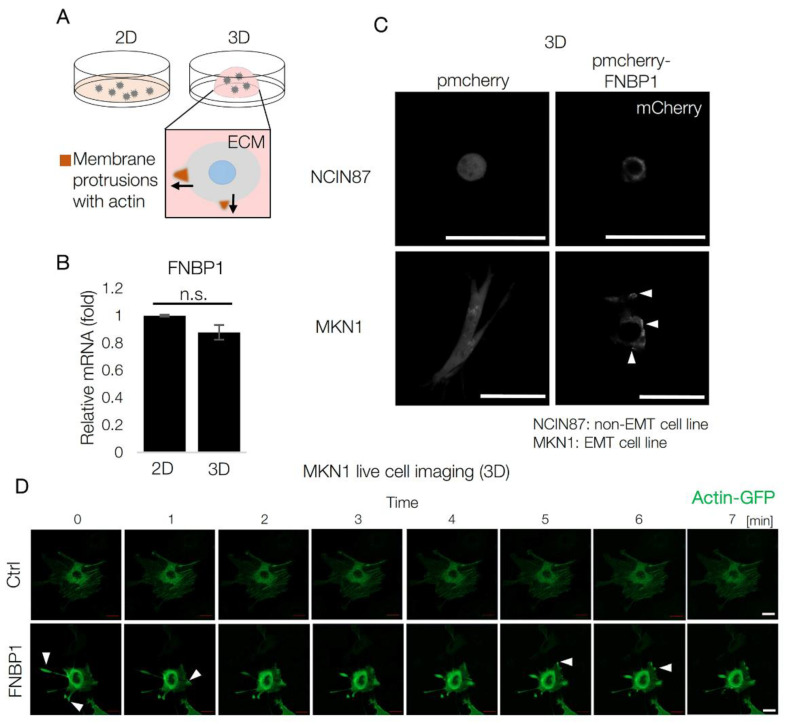
FNBP1 overexpression up-regulates cell motility via increasing membrane protrusions. (**A**) Diagram explaining two-dimensional and three-dimensional culture systems. Cellular movement in dense extracellular matrix, Matrigel matrix, is drawn below with arrows indicating the direction of the movement. (**B**) mRNA expression level in different culture conditions. “n.s.” indicates not significant (*p* > 0.05). (**C**) Confocal image showing localization of mCherry in living cells growing in Matrigel matrix. Cells were transfected with pmCherry or pmCherry-FNBP1 72 h before live-cell imaging with confocal microscopy. Arrows mark the areas enriched with FNBP1. Scale bar: 50 µm. (**D**) Live cell imaging of actin dynamics in FNBP1-overexpressed cells with confocal microscopy. Arrows mark the actin-enriched areas. Scale bar: 20 µm. Data represent mean ± SD.

To investigate the role of FNBP1 in cell motility, we have cultured cells in three-dimensional (3D) conditions (Figure 4A). Cells migrate in various environments with different protrusion mechanisms [33]. Cells migrate with membrane protrusions called filopodia and lamellipodia in 2D. In a thick extracellular matrix, cells form membrane protrusions, such as invadopodia or podosomes, which require remodeling activity of the surrounding matrix [34]. These membrane protrusions at the invading area are enriched with actin filaments [35,36]. Aberrant activation of cell protrusive activity results in increased cell motility, possibly leading to metastatic tumors [37].

In terms of FNBP1, there was no difference in mRNA expression level in 2D and 3D culture conditions (Figure 4B). To explore the subcellular localization of FNBP1, cells were transfected with mCherry-tagged FNBP1 [38], and live-cell imaging was performed on cells growing in a 3D matrix dome. Interestingly, FNBP1 was localized in the barbed ends near the plasma membrane only in MKN1, EMT GC cell line, not NCIN87, non-EMT GC cell line; FNBP1 is shown to have diffuse cytoplasmic localization in NCIN87 (Figure 4C). Indeed, FNBP1-overexpressed cells in the 3D dome showed a more active movement of actin at the membrane protrusions compared to the control (Figure 4D). These results suggest that FNBP1 overexpression induces actin enrichment at the invading area in cells growing in extracellular matrix, indicating its role in the formation of invadopodia or podosomes. 

### 2.5. Sp1 Induces FNBP1

**Figure 5:**
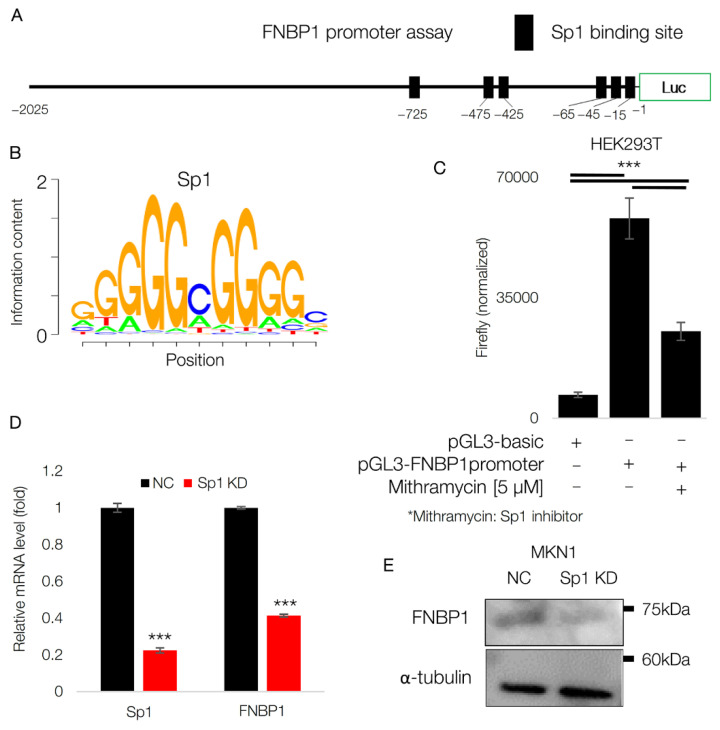
Sp1 induces FNBP1. (**A**) Diagram showing Sp1 motif in FNBP1 promoter. (**B**) Oligonucleotide sequence of Sp1 binding motif. (**C**) Luciferase assay with promoter sequence introduced in (**A**) in HEK293T cells. 5 μM of mithramycin or vehicle of equal volume was treated for 24 h. Values were normalized by total protein amount. (**D**) qRT-PCR result is shown in MKN1 with Sp1 siRNA or scramble siRNA. (**E**) Immunoblot showing the protein level of FNBP1 upon Sp1 knockdown. Data represent mean ± SD. *** *p* < 0.001; two-tailed *t*-test.

Since FNBP1 has a wide range of expression levels across GC cell lines with an abundance in EMT GC cell lines, we next investigated it on the driver transcription factor. First, we analyzed the FNBP1 promoter region. Interestingly, Sp1 binding motif was present abundantly in the region (Figure 5A). Sp1 has long been considered as a basal transcription factor with the main role in the regulation of housekeeping genes [39]. However, Sp1 is drawing attention due to its potential role as a predictor of survival in many types of cancer including GC [18,21,22,40,41,42]. Sp1 binds to GC-rich DNA regions (Figure 5B). We performed a luciferase assay with the promoter region containing approximately 2 kb of a 5′ flanking sequence of FNBP1 (Figure 5C). Interestingly, treatment of Sp1 inhibitor significantly diminished promoter activity of FNBP1, highlighting the importance of Sp1 in FNBP1 expression. Meanwhile, not only therapeutic inhibition of Sp1, but Sp1 knockdown itself decreased the level of FNBP1 in terms of both mRNA and protein (Figure 5D,E). Taken together, Sp1 is a strong driver of FNBP1 expression in EMT-type GC cell lines.

## 3. Discussion

Gastric cancer is adenocarcinoma with great intertumoral heterogeneity [43]. The intertumoral heterogeneity leads to different sensitivity to anti-cancer drugs, highlighting the need for individualized therapy [24]. As an effort, we previously reported the subgroup of GC patients with the worst prognosis and no benefit expected from adjuvant chemotherapy [7]. In addition, the TCGA project and the ACRG defined subtypes of GC introducing GS-type and EMT-type as the most aggressive tumor [4,5]. A common feature among the most aggressive type of GC is that they are prone to metastasis with EMT features. Therefore, it is necessary to identify a key molecule that distinguishes the aggressive subtype from other types with high epithelial integrity and enables high metastatic ability.

Here, we propose FNBP1 as a key to understand the invasiveness in EMT-type GC cells. The role of FNBP1 in GC cells has not been reported yet. Previously, FNBP1 was reported to indicate poor differentiation and invasiveness in breast cancer and bladder cancer [11,16,17]. In this study, we introduce FNBP1 as a distinguisher not between normal tissues and carcinoma, but between EMT and non-EMT type cancer cells in GC, supported by transcriptomic analysis of GC patients and in vitro experiments. 

Furthermore, it is necessary to evaluate the function of FNBP1 in three-dimensional culture conditions where cells are growing in a dense extracellular matrix. Although most cells have been cultured in a monolayer in vitro, there are many limitations primarily due to the fact that 2D cultures do not correctly mimic tissue cells in human patients [44]. Cells migrating on the plane show only transient expression of filopodia unlike cells in 3D which migrate with extensive utilization of actin-rich membrane protrusions [45,46]. The molecular mechanism of the rigorous movement of membrane protrusions is, however, poorly understood [46]. Interestingly, FNBP1 knockdown did not affect cell survival or 2D migration but significantly diminished invasiveness. Indeed, live imaging of MKN1 with FNBP1 overexpression in 3D showed more extensive movement of actin along with localized distribution of FNBP1 near the plasma membrane only in the EMT-type GC cell line.

Since GC cell lines have a wide range of FNBP1 expression across the cell lines compared to other cancer cell lines from different organs, it is important to find the transcription driver. Previously, Sp1 was known to be a negative marker for the survival of GC patients, however, with controversial mechanisms [18,22,47,48]. Here, we first propose the Sp1-FNBP1 pathway as a key to explain the negative role of Sp1 in GC. Further research is required to find whether FNBP1 is the major contributor of Sp1 being the negative prognostic marker with more efforts in elaborating the relationship between Sp1 and FNBP1.

In most cases of GC, surgical management is the first-line treatment option [49,50,51]. However, the recurrence rate still remains high even after the complete resection of the tumor, and patients with recurring GC exhibit extremely low survival rates, which indicates the need for a more thorough understanding of GC [52]. As we indicated, GC is a tumor with great heterogeneity which leads to different clinical outcomes. We have previously discovered the stem-like subtype with the worst prognosis, leaving a question of the molecular mechanisms underlying the aggressiveness of the subtype. As an effort, we, therefore, introduce FNBP1 which controls the invasiveness of the aggressive type of GC cells.

## 4. Materials and Methods

### 4.1. Cell Culture

MKN1, SNU484, SNU601, KATOIII, and NCIN87 cells were cultured in RPMI1640 containing 10% fetal bovine serum (FBS), 2 mM L-glutamine, 100 U/mL penicillin, and 100 µg/mL streptomycin. HS746T was cultured in DMEM containing 10% FBS, 2 mM L-glutamine, 100 U/mL penicillin and 100 µg/mL streptomycin. Cells were cultured in a humidified 5% CO_2_ atmosphere at 37 °C. All cell lines were tested for mycoplasma contamination. For 3D culture, cells were grown in an extracellular matrix dome composed of 50% Matrigel matrix (Corning, Corning, NY, USA) and 50% complete media.

### 4.2. shFNBP1 Cell Line Generation

Five shRNA constructs targeting FNBP1 were selected from the RNAi Consortium (TRCN0000149573, TRCN0000148457, TRCN0000149139, TRCN0000149751, TRCN0000148492). Recombinant lentivirus was produced by co-transfection of pMD2.G, psPAX2, and each construct in 293T packaging cells. MKN1 underwent the selection process with 2 µg/mL of puromycin (Sigma Aldrich, St. Louis, MO, USA) 48 h after produced lentivirus was added to complete media.

### 4.3. Transcriptome Analysis

We downloaded mRNA microarray data of ACRG (GSE62254) and microarray data of Yonsei cohort (GSE13861 and GSE84437) from the Gene Expression Omnibus. RNA sequencing data and clinicopathological variables on the TCGA stomach adenocarcinoma were downloaded from cBioportal. We also extracted the expression level of FNBP1 across the cancer cell lines that originated from different organs through the CCLE database.

### 4.4. mRNA Sequencing 

Total RNA was extracted by using TRIzol reagent (ambion, Austin, Tx, USA). Total RNA concentration was calculated by Quant-IT RiboGreen (Invitrogen, #R11490). To assess the integrity of the total RNA, samples are run on the TapeStation RNA screentape (Agilent, #5067-5576). Only high-quality RNA preparations, with RIN greater than 7.0, were used for RNA library construction. RNA-seq libraries were generated with 1 µg of total RNA by using Illumina TruSeq RNA sample Preparation Kit (Illumina, San Diego, CA, USA). mRNA was purified using poly-T-attached magnetic beads. The libraries were quantified using KAPA Library Quantification kits for Illumina Sequencing platforms according to the qPCR Quantification Protocol Guide (KAPA BIOSYSTEMS, Wilmington, MA, USA) and qualified using the TapeStation D1000 ScreenTape (Agilent, Santa Clara, CA, USA). Indexed libraries were finally submitted to an Illumina NovaSeq, and the paired-end (2 × 100 bp) sequencing was performed by Macrogen Incorporated. 

### 4.5. Invasion Assay

The invasion ability was measured using a 6.5 mm-transwell with 8.0 µm-pore polycarbonate membrane insert (Corning, Corning, NY, USA) after coating with Matrigel (Corning, Corning, NY, USA) diluted to 0.67 µg/µL with serum-free media. Then, the cells (2 × 10^4^/well) were suspended in 200 µL of serum-free medium, and 1000 µL of culture medium with 10% FBS was added into the lower chamber as a chemoattractant. After 8 h, the invading cells were stained with 0.2% crystal violet and photographed under a bright-field microscope (10×). The average number of cells that penetrated the membrane was calculated with ImageJ (NIH) from three randomly selected high-power fields and from two independent experiments.

### 4.6. RNA Extraction and Real-Time Quantitative PCR Analysis

mRNA expression level was analyzed with real-time PCR. Total RNA was isolated from cell lines or organoids and used to synthesize cDNA with random hexamer primers and SuperScript reverse transcriptase II (Invitrogen, Carlsbad, CA, USA). Real-time PCR was conducted with SYBR Green PCR Master Mix (Applied Biosystems, Waltham, MA, USA) using ABI PRISM 7300 RT-PCR system (Applied Biosystems, Waltham, MA, USA). Data were normalized to 36B4 RNA expression level in PCR analysis. The ΔΔ-Ct method was used for quantification (Applied Biosystems, Waltham, MA, USA). The following primers were used for RT-PCR: FNBP1, 5′-TGCAAAGCAACTCAGGAATCT-3’, 5′-TTC ATT TCG TTC AGG TTG GAA-3’; 36B4, 5′-CCT TCT CCT TTG GGC TGG TCA TCC A-3′, 5′-CAG ACA CTG GCA ACA TTG CGG ACA C-3′, Sp1, 5′-CCA TAC CCC TTA ACC CCG-3’, 5′-GAA TTT TCA CTA ATG TTT CCC ACC-3′.

### 4.7. Live Cell Immunofluorescence

MKN1 cells were transfected with pmCherry-C1 with or without FNBP1 insertion (Addgene #27688). Live cells are plated in a µ-Dish (Ibidi, Gräfelfing, Germany) with Matrigel matrix (Corning, Corning, NY, USA). For actin labeling, cells were incubated in the media containing CellLight™ Actin-GFP (Invitrogen) for 1 h before the imaging step. Cells were imaged with an LSM780 microscope (Carl Zeiss, Oberkochen, Germany).

### 4.8. Luciferase Assay

HEK293T cells were cultured into 12-well plates. After a 24-h incubation, the pGL3-basic reporter vector (Addgene, Watertown, MA, USA) carrying the promoter sequence was transfected into GC cells. After 48 h, the luciferase activity was measured by a luciferase reporter system (Promega, Madison, WI, USA). Firefly luciferase activities were normalized to the total protein amount. FNBP1 promoter sequence: chromosome 9:130,043,190-130,045,214, reverse strand. 

### 4.9. Visualization and Analyzation

Visualization and analyzation were based on differentially expressed genes with a fold change greater than two and a *p*-value smaller than 0.05. DAVID (https://david.ncifcrf.gov/summary.jsp (accessed on 22 March 2021)) was performed for KEGG (http://www.genome.jp/kegg (accessed on 22 March 2021)) analysis. Gene set enrichment analysis (GSEA v4.0) was conducted using the FPKM value of RNA-seq data.

### 4.10. siRNA

MKN1 cells were seeded on 6-well plates with complete media for 24 h. Then, 20 nM siRNAs against Sp1 (Santa Cruz, Dallas, TX, USA) and scramble siRNA (Bioneer, Daejeon, Korea) were transfected via electroporation (2 pulses, 1100 volt, and 20 msec). Protein and mRNA samples are prepared 72 h after the transfection.

### 4.11. Statistical Analyses

Statistical analysis was performed by Student’s *t*-test when two groups were analyzed. The *p*-values were adjusted with Benjamini–Hochberg false discovery rate method in analyzing microarray data of human patients. The *p*-values below 0.05 were marked as statistically significant (* *p* < 0.05, ** *p* < 0.01, *** *p* < 0.001). All values were indicated by means with standard deviation.

## 5. Conclusions

Here, we showed that FNBP1 is up-regulated in EMT-type GC patients. Consistent with the transcriptomic findings in human patients, FNBP1 expression level was higher in EMT-type GC cell lines. To find the role of FNBP1, we performed loss-of-function test, which resulted in the loss of invasive ability, specifically. Also, FNBP1 overexpression led to rigorous motility of cells, growing in dense extracellular matrix, via increasing membranous protrusions. Therefore, we propose FNBP1, with Sp1 as the transcription driver, as a novel therapeutic target for EMT-type GC.

## Figures and Tables

**Table 1:** KEGG pathway analysis in MKN1 with or without FNBP1 knockdown.

KEGG	*p*-Value	Bonferroni	FDR
Axon guidance	4.9 × 10^−6^	0.00136	0.00017
Cell adhesion molecules	2.6 × 10^−5^	0.00732	0.00072
Focal adhesion	0.00028	0.07679	0.00427
Cell cycle	1	1	1

## Data Availability

Fastq files for shFNBP1 and shControl cell lines were uploaded to NCBI GenBank.
